# Alpha-linolenic acid protects against heatstroke-induced acute lung injury by inhibiting ferroptosis through Nrf2 activation

**DOI:** 10.1080/13510002.2025.2538294

**Published:** 2025-07-27

**Authors:** Lin Wang, Jiamin Ma, Zhaozheng Li, Xinru Zhao, Ying Chen, Pei Wang, Yi Li, Yuwei Chen, Xuanqi Yao, Liangfang Yao, Jinbao Li

**Affiliations:** a School of Anesthesiology, Shandong Second Medical University, Weifang, People’s Republic of China; b Department of Anesthesiology, Shanghai General Hospital, Shanghai Jiao Tong University School of Medicine, Shanghai, People’s Republic of China

**Keywords:** Heatstroke, alpha-linolenic acid, Nrf2, ferroptosis, acute lung injury, SLC7A11, GPX4, lipid peroxidation

## Abstract

Heatstroke (HS)-induced acute lung injury (ALI) has high morbidity and mortality with no specific therapies. Ferroptosis, a form of programmed cell death driven by lipid peroxidation due to reduced Glutathione Peroxidase 4 (GPX4) activity, is closely linked to HS-induced ALI. This study investigated the effect of alpha-linolenic acid (ALA), a plant-derived ω-3 fatty acid, on ferroptosis in a mouse model of HS-induced ALI. Histopathology analysis found that ALA can attenuate lung injury and improve the 7-day survival rate in mice with HS-induced ALI. In addition, ALA significantly reduced the levels of reactive oxygen species (ROS) and malondialdehyde (MDA), while increasing the level of antioxidant glutathione (GSH). Further analysis showed that ALA upregulated the levels of SLC7A11 and GPX4 by promoting the nuclear translocation of Nrf2. This led to increased GSH synthesis but reduced ROS accumulation, which in turn suppressed ferroptosis and protected the mice against HS-induced ALI. Additionally, the protective effect of ALA was found to be diminished in *Nrf2*-deficient mice. In summary, ALA inhibits ferroptosis in macrophages by activating the Nrf2/SLC7A11/GPX4 pathway and attenuates HS-induced ALI.

## 1. Introduction

Heatstroke (HS) is characterized by extreme hyperthermia, usually exceeding 40.5°C along with a thermoregulatory imbalance [1]. This condition can occur when the body is exposed to high temperatures and high humidity for an extended period. It is often accompanied by abnormalities of the central nervous system and multiple organ dysfunction syndrome (MODS), which can be a serious threat to the patient's life [2]. The pathophysiological mechanism of HS has not been fully elucidated. Most scholars believe that HS is the result of complex interactions between direct cellular damage and the systemic inflammatory response induced by heat exposure, which in turn leads to MODS [3]. The clinical features of HS-induced acute lung injury (ALI) include inflammatory cell infiltration, pulmonary edema, arterial hypoxemia, and vascular endothelium and alveolar epithelium damage, leading to impaired lung function. There is increasing evidence that ALI is associated with inflammatory injury, disseminated intravascular coagulation, oxidative stress, cellular pyroptosis and ferroptosis [4]. However, the exact mechanism of HS-induced ALI remains unclear. Economical, safe, and effective means of prevention and treatment have yet to be developed.

Ferroptosis is a unique mode of iron-dependent cell death driven by phospholipid peroxidation. It is regulated by a variety of cellular metabolic pathways, including redox homeostasis, iron metabolism, mitochondrial activity, amino acid, lipid, and glucose metabolism [5]. It is established that ferroptosis is involved in the development and regression of many diseases, including cancer [6], neurodegenerative diseases [7], cardiovascular diseases [8], lung diseases [9] and ischemia-reperfusion diseases [10]. Notably, ferroptosis closely correlates with the pathogenesis of lung injuries [11], such as sepsis-induced ALI [12], ischemia-reperfusion-induced ALI [13], and hyperoxic ALI [14]. The pathogenesis of HS-induced ALI, especially in the presence of ferroptosis, remains elusive.

Alpha-linolenic acid (ALA), an essential fatty acid, has pharmacological activities, such as anti-inflammatory and antioxidant effects [15]. Our previous study induced alveolar macrophage pyroptosis using neutrophil extracellular traps (NETs) in a mouse model of sepsis-induced ALI and found that ALA attenuated the pyroptosis by inhibiting pyrin inflammasome activation [16]. However, the effect of ALA in HS-induced ALI with ferroptosis and the underlying molecular mechanism are still unknown. This study established a mouse model of HS-induced ALI and explored the role of ALA in ferroptosis during HS-induced ALI.

## 2. Materials and methods

### 2.1. Preparation of animals

Male C57BL/6 mice aged 6–8 weeks and weighing 18–24 g were purchased from Jihui Laboratory Animal Care Co., Ltd. (Shanghai, China).

**Figure 1.**
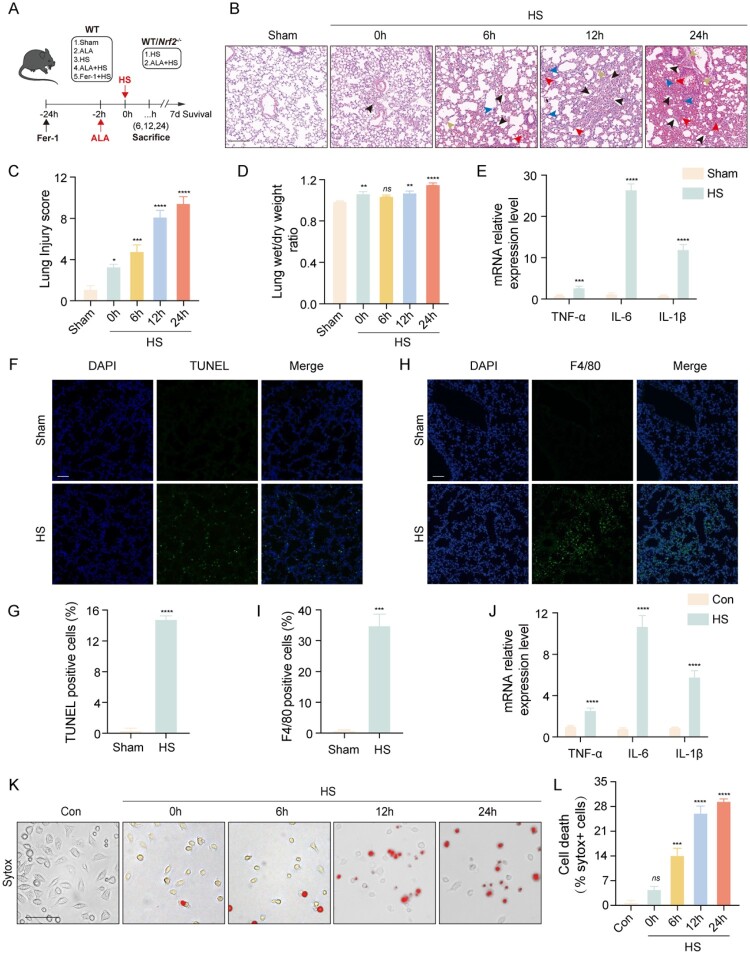
HS induces ALI in mice and macrophage death. (A) Experimental strategy for HS mice in this study. (B) Representative images showing H&E staining of lung sections from different time points. Yellow arrowheads indicate edema, red indicates erythrocyte exudation, blue indicates inflammatory cell infiltration, and black indicates alveolar septa thickening (scale bar = 200 μm). (C) Histopathological scoring of lung injury. (D) Wet/dry ratio of the lung tissue. (E) qRT-PCR analysis of TNF-α, IL-6 and IL-1β in the lung tissue of mice in each group (n ≥ 3). (F, G) Images and semi-quantification of TUNEL and DAPI immunofluorescence staining in the lung tissue of mice (scale bar = 50 μm). (H, I) Images and semi-quantification of immunofluorescence staining for F4/80 and DAPI in the lung tissue of mice (scale bar = 100 μm). (J) qRT-PCR analysis of TNF-α, IL-6, and IL-1β in BMDMs from each group (n ≥ 3). (K, L) The dead cells were detected using the SYTOX fluorescence staining images (scale bar = 100 μm) and semi-quantification analysis in BMDMs. Data are presented as mean ± SD and assessed by one-way ANOVA. *P < 0.05, ** *P* < 0.01, ***P < 0.001.

*Nrf2* (Nfe2l2)*^–/–^* mice were generated by Cygen Biosciences (Suzhou, China) and bred by the Animal Laboratory of the First People’s Hospital affiliated with Shanghai Jiaotong University. All animal experiments were conducted in accordance with the Guidelines for the Care and Use of Laboratory Animals and approved by the Animal Ethics Committee of Shanghai First People's Hospital (No. 2023AW055). The mice were randomly divided into five groups (n = 8) according to the experimental design: (1) the Sham group, (2) the ALA (1500 mg/kg; L2376, Sigma-Aldrich) group, (3) the HS (39.5 ± 0.2°C; 65 ± 5% RH) group, (4) the ALA + HS group, and (5) the Ferrostatin-1 (Fer-1, 10 mg/kg; HY-100579, MCE) + HS group. The HS model was established at high ambient temperature (39.5 ± 0.2°C) and high humidity (65 ± 5% RH). To determine whether ALA exerts a protective effect through the activation of Nrf2, the mice were divided into four groups (n = 12): (1) the WT-HS group, (2) the WT-ALA + HS group, (3) the *Nrf2^–/–^*-HS group, and (4) the *Nrf2^–/–^*-ALA + HS group. Mice received an intraperitoneal injection of Fer-1 (10 mg/kg) 24 hours before HS and ALA (1500 mg/kg) 2 hours before HS. All mice were anesthetized with 5% isoflurane 24 h after successful model establishment to obtain blood and lung tissue (Figure 1A).

### 2.2. HS model establishment

The day before heat exposure, 6- to 8-week-old male C57BL/6 mice were placed in an artificially temperature-controlled climate chamber at 25 ± 2°C to accommodate incubator noise. The HS model was established by placing the mice in a high ambient temperature (39.5 ± 0.2°C) and high humidity (65 ± 5% RH). Core body temperature (Tc) was measured via a rectal thermocouple (BW-TH1101; Biowill, Shanghai, China) and monitored every 15 min and changed every 5 min when Tc rose to 41°C. In order to clarify the different levels of severity of HS, we categorized it as mild (Tc 42–42.5°C), moderate (Tc 42.5–43°C), and severe (Tc > 43°C) [17]. The survival rates of the HS-treated mice in each category are shown below (Figure S1A). We developed a mouse model of moderate HS, and successful modeling was defined as Tc of 42.5°C or the appearance of neurological symptoms, such as hemiparesis and loss of the turning reflex [18,19]. After the successful establishment of the model, the mice were immediately returned to their original cages at an ambient temperature of 25°C, with free access to food and water. The control group underwent the same process without heat stress exposure.

### 2.3. Histopathological analysis

Fresh lung tissue was promptly collected, fixed in 4% paraformaldehyde (PFA), and subsequently embedded in paraffin. The paraffin-embedded tissue blocks were sliced into 4-µm-thick sections, stained with hematoxylin and eosin (H&E), and photographed under a light microscope. The assessment was performed by two professionals who were unaware of the experimental design and was based on the Smith Lung Injury Score, which consists of four aspects: alveolar and interstitial hemorrhage; alveolar and interstitial inflammation; pulmonary edema; pulmonary atelectasis and hyaline membrane formation [20]. The score was determined by all Smith Score components in the randomized field of view (n = 6 per group). The scoring system is defined as follows: a score of 0 indicates no lesions in the lung tissue, a score of 1 indicates that less than 25% of the lung tissue has lesions, a score of 2 indicates that 25–50% of the lung tissue shows lesions, a score of 3 indicates that 50–75% of the lung tissue is affected by lesions, and a score of 4 indicates that more than 75% of the lung tissue has lesions. The overall lung injury score is the sum of the four items mentioned above.

### 2.4. Lung wet/dry ratio

The fresh lung tissue was drained and weighed to obtain its wet weight, then placed in a drying oven at 60°C for 48 h to obtain the dry weight. The wet/dry weight ratio was calculated to indicate the severity of pulmonary edema.

### 2.5. TUNEL staining

Cell death was detected via a One Step TUNEL Apoptosis Detection Kit (C1086, Beyotime). Green fluorescent-positive cells were detected using fluorescence microscopy, and quantitative analysis was conducted with ImageJ software.

### 2.6. Biochemical reagent kit

Serum iron (A039-1-1), malondialdehyde (A003-1-2) and GSH (A006-2-1) were assessed using biochemical kits from Nanjing Jiancheng Bioengineering Institute in Nanjing, China.

### 2.7. Cell culture and treatment

Bone marrow-derived macrophages (BMDMs) were isolated according to the method described in a previous study [21]. Adult mice were humanely sacrificed and subsequently immersed in 75% alcohol. The bone marrow was collected from femurs and tibiae and flushed with sterile precooled 1640 medium (Gibco). Erythrocytes were lysed by adding erythrocyte lysate (C3702, Beyotime). The final harvested cells were cultured in 1640 complete medium containing 10% fetal bovine serum (FBS, Gibco), 50 µg/ml penicillin/streptomycin (C100C5, NCM), and 10 ng/ml recombinant macrophage colony-stimulating factor (M-CSF, 315-02, PeproTech). The medium was changed with fresh medium every other day. The cells were used for subsequent experiments until they reached maturity on the seventh day. The following groups of BMDMs were treated according to the experimental design: the Control (Con) group, the HS group, the ALA group (ALA with a final concentration of 100 nM), the ALA + HS group, the Fer-1 + HS group (Fer-1 with a final concentration of 10 µM), and the N-acetylcysteine (NAC) + HS group (NAC with a final concentration of 2.5 mM). Cellular thermal stimulation: The heat stress (HS) group was subjected to 43°C heat exposure for 90 min in a thermal incubator. ALA, NAC, and Fer-1 were administered as pretreatments one hour before heat stress. The control group received no heat stress or drug treatment.

### 2.8. Reactive oxygen species (ROS) detection

ROS were detected using a reactive oxygen species assay kit (S0033S, Beyotime). Photographs were subsequently taken with a confocal microscope (Leica, Germany), and quantitative analysis was performed utilizing ImageJ software.

### 2.9. BODIPY C11 assay

Reactive oxygen species were detected in cells and cell membranes using a lipid peroxidation sensor (D3861, Thermo). Detection was carried out through fluorescence microscopy, where oxidizing cells exhibited green fluorescence while reducing cells displayed red fluorescence. A quantitative analysis was performed using ImageJ software.

### 2.10. Quantitative real-time PCR

BMDMs and lung tissue were lysed using TRIzol, followed by purification of RNA through sequential addition of trichloromethane, isopropanol, and 75% ethanol. RNA concentration was then measured. PCRs were conducted according to the instructions provided with the HyperScript III RT SuperMix for the qPCR kit, and the experiment was repeated at least three times. Statistical analysis was performed via GraphPad Prism software, and the primer sequences for real-time quantitative PCR are shown in Table 1.

**Table 1.** Sequences of the quantitative real-time PCR primers.

Gene	Forward Primer sequence 5’−3’	Reverse Primer sequence 5’−3’	Accession	
m18S	TTCCGATAACGAACGAGACTCT	TGGCTGAACGCCACTTGTC	NR_003278.3	
mGAPDH	AGGTCGGTGTGAACGGATTTG	TGTAGACCATGTAGTTGAGGTCA	NM_008084.4	
mIL-1β	GCAACTGTTCCTGAACTCAACT	ATCTTTTGGGGTCCGTCAACT	XM_006498795.5	
mTNFα	CCCTCACACTCAGATCATCTTCT	GCTACGACGTGGGCTACAG	NM_013693.3	
mIL-6	TAGTCCTTCCTACCCCAATTTCC	TTGGTCCTTAGCCACTCCTTC	NM_031168.2	
mGPX4	GATGGAGCCCATTCCTGAACC	CCCTGTACTTATCCAGGCAGA	NM_001037741.4	
mSLC7A11	GGCACCGTCATCGGATCAG	CTCCACAGGCAGACCAGAAAA	XM_006500776.5	
mNrf2	TCTTGGAGTAAGTCGAGAAGTGT	GTTGAAACTGAGCGAAAAAGGC	NM_010902.5	

### 2.11. Immunofluorescence staining

BMDMs were inoculated in 24-well plates covered with sterile coverslips. When differentiated and matured, they were treated according to the experimental protocol. The treated cells were fixed in 4% paraformaldehyde, infiltrated with 0.5% Triton X-100, blocked using 1% bovine serum albumin (BSA), and incubated with primary antibodies overnight at 4°C. The primary antibodies used were GPX4 (1:2000, ab252833, Abcam) and Nrf2 (1:2000, 20733, CST). After washing the cells three times, they were incubated with secondary antibodies. The cells were then coincubated with DAPI for the patching process. The cells were incubated with SYTOX (1:2000, Invitrogen, S7020) for 15 min at room temperature in the dark for SYTOX staining. Images were captured using a Leica SP8 confocal microscope, and quantitative analysis was conducted with ImageJ software.

### 2.12. Western blot analysis

BMDMs and lung tissue were lysed using RIPA lysis buffer (WB3100, NCM) and then centrifuged at 12000rpm for 10 min at 4°C. The protein concentration was measured using a BCA protein assay kit (WB6501, NCM). Next, the protein lysates was separated via 10% SDS-PAGE and transferred them onto a PVDF membrane. The membrane was blocked with Blot Blocking Buffer (P30500, NCM) for 15 min at room temperature, followed by three washes with TBST. After that, the membrane was incubated with primary antibody at 4°C overnight. After three washes with TBST, the membrane was incubated with the secondary antibody at room temperature for 2 h, followed by three additional washes with TBST. Finally, the membrane was developed using Omni-ECL™ with the Bio-Rad ChemiDoc XRS system and Image Lab software, and the protein bands were analyzed with ImageJ software. Information regarding the antibodies, dilution ratios, and sources can be found in Table 2.

**Table 2.** Western blot antibody information.

Antibody	Catalog Number	Dilution Ratio	Manufacturer	Molecular Weight
GPX4	ab252833	1:2000	Abcam	17KDa
FTH	ab183781	1:1000	Abcam	23KDa
SLC7A11	DF12509	1:1000	Affinity	55KDa
ASCL4	66617-1-Ig	1:1000	Proteintech	70KDa
Nrf2	20733	1:2000	CST	100KDa
HO-1	ab189491	1:2000	Abcam	33KDa
β-actin	ab179467	1:1000	Abcam	43KDa

### 2.13. Statistical analyses

To compare two groups of normally distributed data, we utilized a two-tailed Student's t test. For multiple comparisons, we employed one-way ANOVA. For survival analysis, we conducted Kaplan–Meier analysis. Differences were considered statistically significant when the *P*-value was less than 0.05.

## 3. Results

### 3.1. HS induces ALI in mice and macrophage death

HS can lead to early multiorgan damage, resulting in increased morbidity and mortality [2]. We examined the pathological sections of various organs by H&E staining at different time points (0, 2, 6, 12, and 24 h) post-modeling. Our findings indicated that the HS mice experienced multiorgan failure (Figure S1B), with significant damage observed in the lungs. H&E staining revealed that edema (yellow arrowheads), hemorrhage (red arrowheads), inflammatory cell infiltration (blue arrowheads), and alveolar wall thickening (black arrowheads) progressively worsened in the lung tissue of the HS mice over time (Figure 1B). The lung damage score for the HS 24 h group was significantly higher than that of the other groups, and the wet/dry ratio of the lungs in the HS 24 h group indicated the most severe lung edema (Figures 1C, D). Therefore, we decided to use the HS 24 h model for the subsequent study. We observed that the mRNA levels of TNF-α, IL-6, and IL-1β in the lung tissue of the HS group were significantly higher than those in the sham group (Figure 1E). Additionally,

TUNEL staining indicated a notable increase in cell death in the lungs of the mice from the HS group (Figures 1F, G). Moreover, a significant increase in macrophage infiltration in the lung tissue of the HS group was confirmed through immunostaining for the macrophage marker F4/80 (Figures 1H, I). Afterward, we prepared an HS cell model in vitro at a high ambient temperature (43°C). Further immunofluorescence staining of macrophages showed many dead cells in the HS group (Figures 1K, L). The mRNA levels of TNF-α, IL-6, and IL-1β were significantly higher in the HS group compared to the Con group (Figure 1J). In summary, HS can lead to macrophage infiltration and cause ALI in mice, with the most significant effects observed 24 h after HS.

### 3.2. ALA ameliorates HS-induced ALI and inhibits macrophage death

**Figure 2.**
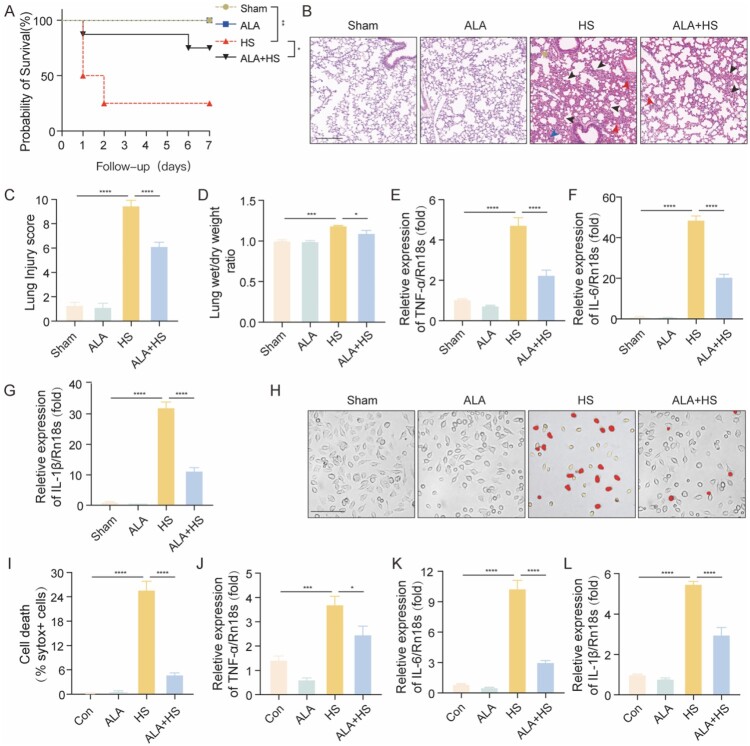
ALA ameliorates HS-induced ALI and inhibits macrophage death. (A) Kaplan–Meier survival analysis for each group (n = 8 per group). (B) Representative images showing H&E staining of lung sections. Yellow arrowheads indicate edema, red indicates erythrocyte exudation, blue indicates inflammatory cell infiltration, and black indicates alveolar septa thickening (scale bar = 200 μm). (C) Lung injury scores. (D) Wet/dry weight ratios of the lungs for each group. (E-G) qRT-PCR analysis of TNF-α, IL-6, and IL-1β mRNA in the lung tissue of mice in each group (n ≥ 3). (H, I) Dead cells were detected using the SYTOX fluorescence staining (scale bar = 100 μm) and semi-quantification analysis in BMDMs. (J-L) qRT-PCR analysis of TNF-α, IL-6, and IL-1β mRNA in BMDMs in each group (n ≥ 3). Data are presented as mean ± SD and assessed by one-way ANOVA. *P < 0.05, ** *P* < 0.01, ***P < 0.001.

Our previous study found that ALA has a significant therapeutic effect on sepsis-induced ALI [22]. To investigate the effect of ALA on HS-induced ALI in mice, we calculated the survival rate of HS mice after administering ALA. The survival rate was significantly higher in the ALA + HS group compared to the HS group (Figure 2A). We observed that lung injury improved in the ALA + HS group compared to the HS group from the H&E stain, showing significantly reduced inflammatory cell infiltration and erythrocyte exudation (Figures 2B, C). The lung wet/dry ratio indicated that pulmonary edema was significantly lower in the ALA + HS group compared to the HS group (Figure 2D). The levels of mRNA for the inflammatory factors TNF-α, IL-6, and IL-1β were significantly lower in the ALA + HS group compared to the HS group in the lung tissue of the mice (Figures 2E-G). Sytox staining indicated that the ALA + HS group had a lower number of dead cells compared to the HS group (Figures 2H-I). The mRNA levels of TNF-α, IL-6, and IL-1β in macrophages from the ALA + HS group were significantly lower than those in the HS group, as shown in the PCR assay results (Figures 2J-L). These results suggest that ALA ameliorates HS-induced ALI and inhibits macrophage death.

### 3.3. Ferroptosis occurs in HS-induced ALI and can be inhibited by ALA

**Figure 3.**
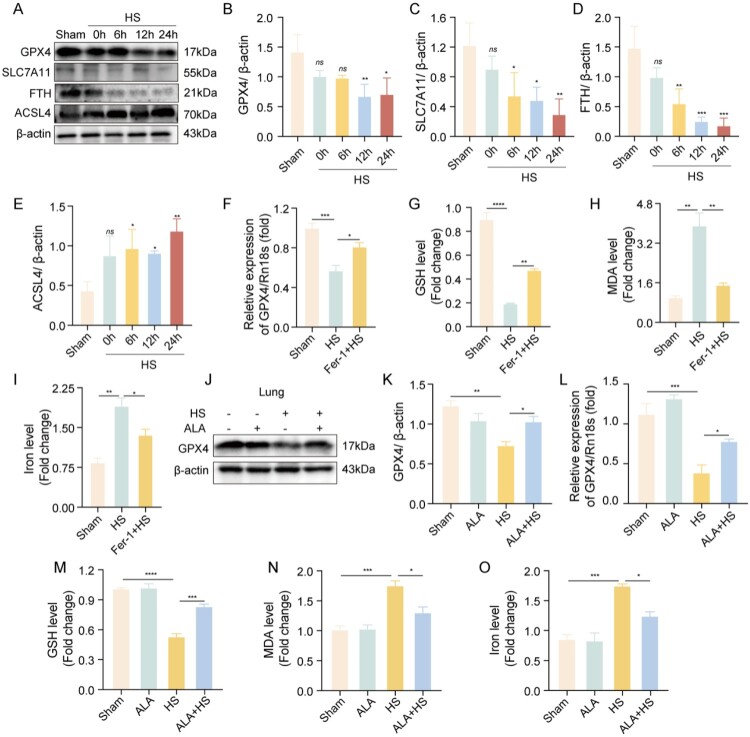
Ferroptosis occurs in HS-induced ALI and can be inhibited by ALA. (A-E) Representative western blot and quantification analyses of GPX4, SLC7A11, FTH, and ACSL4 protein levels in the lung tissue of mice at different time points after HS. (F) qRT-PCR analysis of GPX4 mRNA in the lung tissue of mice in each group (n ≥ 3). (G-I) GSH, MDA, and iron levels in the lung tissue of mice in each group (n ≥ 3). (J, K) Representative western blot and quantification analyses of GPX4 protein levels in the lung tissue of mice in each group (n ≥ 3). (L) qRT-PCR analysis of GPX4 mRNA in the lung tissue of mice in each group (n ≥ 3). (M–O) GSH, MDA, and iron levels in the lung tissue of mice in each group (n ≥ 3). Data are presented as mean ± SD and assessed by one-way ANOVA. *P < 0.05, ** *P* < 0.01, ***P < 0.001.

Ben Lu et al. reported on the possible role of pyroptosis, apoptosis, and necrosis in the pathogenesis of HS [19]. However, the role of ferroptosis in the development of HS-induced ALI is still unclear. Therefore, we focused on whether ferroptosis is involved in the pathophysiological process of HS-induced ALI in mice. We observed a significant decrease in the levels of the anti-ferroptosis proteins GPX4, FTH, and SLC7A11 and a significant increase in the pro-ferroptosis protein ACSL4 in the HS group compared to the sham group (Figures 3A-E). The production level of GSH increased, while iron and malondialdehyde (MDA) levels decreased in the HS + Fer-1 (a GPX4 agonist) group compared to the HS group (Figures 3F–I). The findings indicate that ferroptosis is an important factor in the HS model. In the ALA + HS group, we observed a phenomenon resembling that seen in the Fer-1 + HS group. In comparison to the HS group, the ALA + HS group showed higher protein and mRNA levels of GPX4 (Figures 3J-L) and increased GSH production (Figure 3M). Additionally, the ALA + HS group had lower iron and MDA levels compared to the HS group (Figures 3N, O). These results suggest that ferroptosis occurs in HS-induced ALI and can be inhibited by ALA.

### 3.4. ALA inhibits HS-induced ferroptosis in macrophages

**Figure 4.**
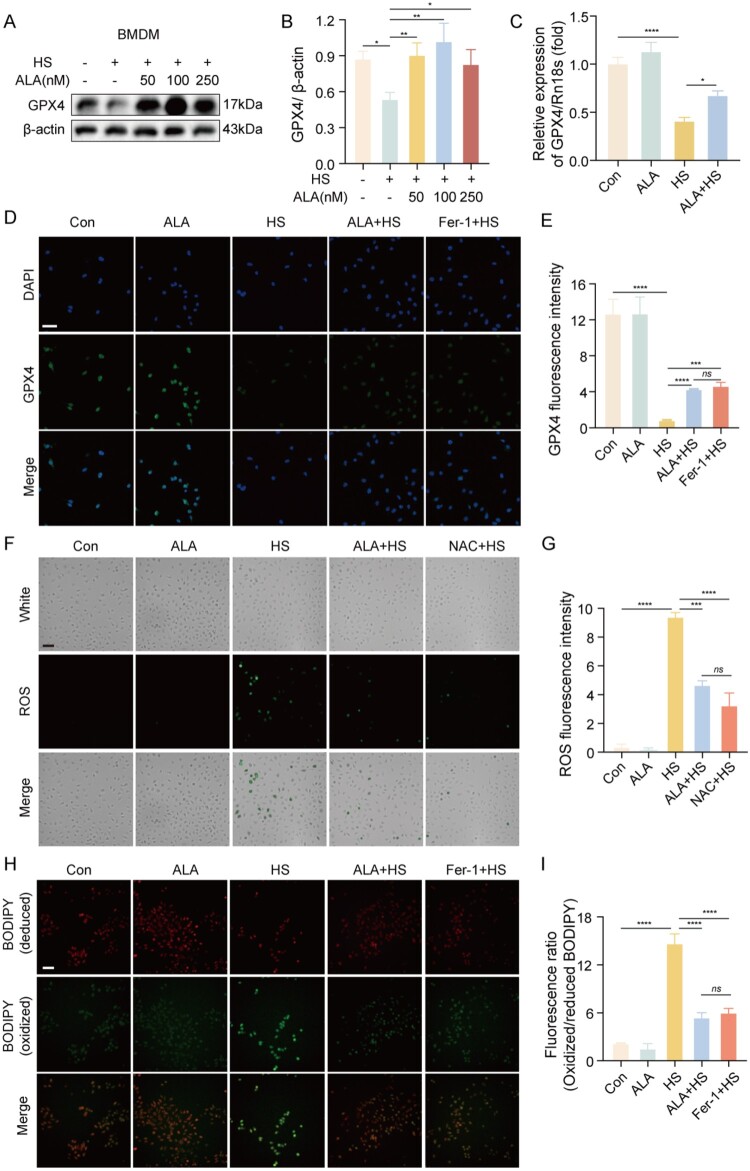
ALA inhibits HS-induced ferroptosis in macrophages. (A, B) Representative western blot and quantification analyses of GPX4 protein levels in BMDMs (n ≥ 3) after pretreatment with different concentrations of ALA (0, 50, 100, 250 nM). (C) mRNA levels of GPX4 in BMDMs in each group (n ≥ 3). (D, E) Representative immunofluorescence images and semi-quantification analysis of GPX4 (green) staining and cell nuclei (in blue) in BMDMs (scale bar = 25 μm, n ≥ 3). (F, G) Intracellular ROS was measured using the DCFH-DA fluorescent probes and semi-quantification analysis in BMDMs (scale bar = 100 μm, n ≥ 3). (H, I) LPO was measured using the BODIPY 581/591 C11 fluorescent probes and semi-quantification analysis in BMDMs (scale bar = 100 μm, n ≥ 3). Data are presented as mean ± SD and assessed by one-way ANOVA. *P < 0.05, ** *P* < 0.01, ***P < 0.001.

To further investigate the effect of ALA on HS-induced ferroptosis, we pretreated macrophages with ALA for one hour. We tested various concentrations of ALA (0, 50, 100, and 250 nM) to determine whether ALA could inhibit HS-induced ferroptosis in macrophages and to identify the optimal dosage. The results showed that the expression of GPX4 was most significantly upregulated at an ALA concentration of 100 nM (Figures 4A, B). Additionally, we observed that ALA increased the mRNA levels of GPX4 in HS-induced BMDMs (Figure 4C). This finding was further confirmed by immunofluorescence assay, which indicated that GPX4 was lower in the HS group compared to the Con group. In contrast, the addition of ALA or Fer-1 resulted in a significant increase in GPX4 fluorescence intensity compared to the HS group (Figures 4D, E). To further understand the mechanism, we used NAC, a reactive oxygen species (ROS) scavenger, which can neutralize the production of ROS. The fluorescence images indicated that the ROS level was significantly elevated in the HS group. However, the ROS level in the ALA + HS group was lower than that in the HS group, similar to the results observed in the NAC group (Figures 4F, G). Immunofluorescence staining with Bodipy C11 revealed that oxidizing cells (red) fluorescence were higher while reducing cells (green) fluorescence were lower in the HS group than in the Con group. After the addition of ALA or Fer-1, reducing cells (green) fluorescence were found to be greater than in the HS group (Figures 4H, I). These results suggest that ALA treatment significantly inhibits the production of ROS and lipid peroxidation (LPO), thereby reducing HS-induced ferroptosis in the macrophages.

### 3.5. ALA inhibits HS-induced ferroptosis in macrophages through the activation of Nrf2

**Figure 5.**
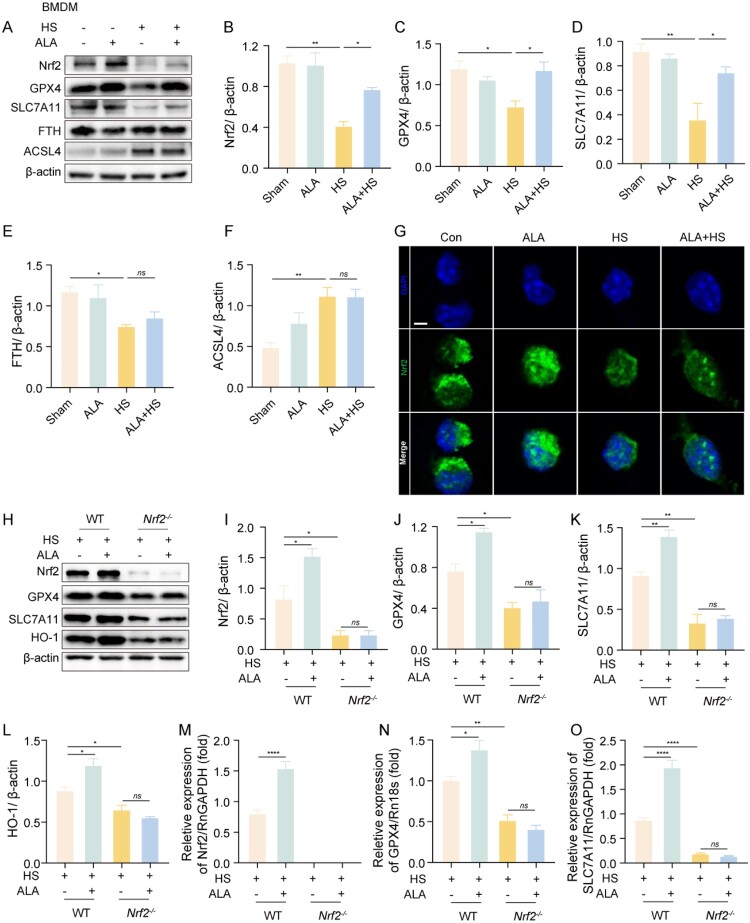
ALA inhibits HS-induced ferroptosis in macrophages through the activation of Nrf2. (A-F) Representative western blot and quantification analyses of Nrf2, GPX4, SLC7A11, FTH, and ACSL4 protein levels in BMDMs. (G) Representative immunofluorescence images of Nrf2 (green) staining and cell nuclei (in blue) in BMDMs (scale bar = 2.5 μm). (H-L) Representative western blot and quantification analyses of Nrf2, GPX4, SLC7A11, and HO-1 protein levels in BMDMs from WT and *Nrf2^–/–^* mice. (M–O) qRT-PCR analyses of the relative mRNA levels of Nrf2, GPX4, and SLC7A11 in BMDMs from WT and *Nrf2^–/–^* mice (n ≥ 3). Data are presented as mean ± SD and assessed by one-way ANOVA. *P < 0.05, ** *P* < 0.01, ***P < 0.001.

After confirming that ALA could inhibit HS-induced macrophage ferroptosis, we sought to further investigate the specific mechanism involved. We observed that the levels of the antioxidant proteins Nrf2, GPX4 and SLC7A11 were significantly lower in the HS group compared with the Con group in HS-induced macrophage. In contrast, these levels were notably increased following ALA administration (Figures 5A-D). Meanwhile, the levels of the iron-autophagy-related protein FTH and the ferroptosis-promoting protein ACSL4 showed no significant difference in the HS + ALA group compared with the HS group (Figures 5A, E-F). Immunofluorescence images indicated that ALA increased Nrf2 expression and its nuclear entry-related transcription (Figure 5G). To further validate the role of Nrf2 in vitro, we generated Nrf2 knockout mice and extracted BMDMs for subsequent experiments (Figure S2). Western blot analysis showed that the expression levels of GPX4, SLC7A11, and HO-1 were lower in the BMDMs of the *Nrf2^–/–^* mice in the HS group compared to those in the wild-type (WT) mice. Furthermore, these differences were not improved by the administration of ALA (Figures 5H-L). In the HS group of *Nrf2^–/–^* mice, the mRNA levels of GPX4 and SLC7A11 were lower compared to the HS group of WT mice, and there was no observable improvement after the administration ALA (Figures 5M–O). These findings suggest that ALA may inhibit HS-induced ferroptosis in macrophages through activation of the Nrf2/SLC7A11/GPX4 axis.

### 3.6. Knockout of Nrf2 exacerbates HS-induced ALI and is not reversed by ALA

**Figure 6.**
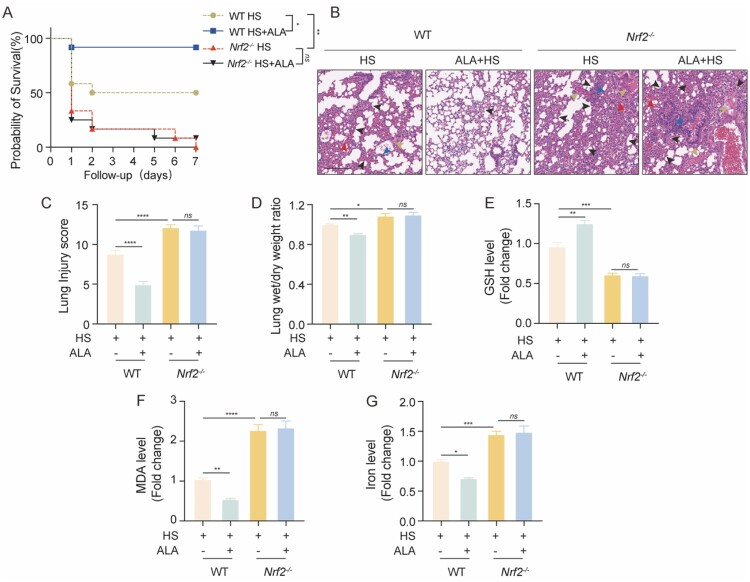
Knockout of Nrf2 exacerbates HS-induced ALI and is not reversed by ALA. (A) Kaplan–Meier survival analysis for each group (n = 12 per group). (B) Representative images showing H&E staining of lung sections. Yellow arrowheads indicate edema, red indicates erythrocyte exudation, blue indicates inflammatory cell infiltrate, and black indicates alveolar septa thickened (scale bar = 200 μm). (C) Lung injury scores. (D) Wet/dry weight ratios of the lungs. (E-G) GSH, MDA, and iron levels in the lung tissue of *Nrf2^-/-^* and WT mice (n ≥ 3). Data are presented as mean ± SD and assessed by one-way ANOVA. *P < 0.05, ** *P* < 0.01, ***P < 0.001.

To further clarify the role of Nrf2 in HS-induced mice, we established the *Nrf2^–/–^* HS model (Figure S2). Our findings indicated that in the HS group, the *Nrf2^–/–^* mice had a higher mortality rate compared to the WT mice, and the administration of ALA did not significantly improve the survival rates of the HS-induced *Nrf2^–/–^* mice (Figure 6A). Additional assessments, including H&E staining, lung injury scores, and lung wet/dry ratios, showed that tissue damage was significantly higher in the *Nrf2^–/–^* mice than in the WT mice induced by HS. However, the administration of ALA did not significantly alleviate lung injury in the HS-induced *Nrf2^–/–^* mice (Figures 6B-D). We also investigated the levels of GSH, MDA and iron in the lung tissues of the mice. The results demonstrated that, compared to WT mice, *Nrf2^–/–^* mice in the HS group exhibited increased accumulation of MDA and iron, as well as decreased GSH production in the lung tissue. These changes were not significantly improved by the administration of ALA (Figures 6E-G). Overall, these findings suggest that ALA alleviates HS-induced ALI in a Nrf2 activation-dependent manner.

## 4. Discussion

The incidence of HS is observed increasing with global warming [23]. Patients with HS typically experience sepsis-like responses, including a systemic inflammatory response (SIRS), disseminated intravascular coagulation (DIC) and multiple organ dysfunction syndrome (MODS) [24]. In this study, a mouse model of moderate-HS was constructed. Consistent with previous research, the model was observed with multiple organ damage, inflammatory cell infiltration and thrombosis [17]. The lungs are affected early in HS. This study found that ALI developed after HS modeling. Additionally, the ALI was observed the most severe at 24 h, accompanied by increased inflammatory factors and macrophage infiltration. This was further confirmed by in vitro experiments. Cell death is of great importance to mammalian development, homeostasis, and disease. Ferroptosis is a newly described form of programmed cell death, characterized by the absence of selenoperoxidase Glutathione Peroxidase 4 (GPX4) or reduced GPX4 activity. It is a unique cell death pathway driven by lipid peroxidation (LPO) [25], and it is different from other forms of regulated cell death, including apoptosis, autophagy, necrosis, and pyroptosis in morphology, biochemistry, and genetics. Ferroptosis correlates to increased mitochondrial potential and decreased membrane density and volume, which are different from necrosis (swelling of cytoplasm, rupture of plasma membrane), apoptosis (chromatin condensation and margination), and autophagy (double-membrane enclosed vesicles) [26,27]. Oxidative stress affects mitochondrial function, iron metabolism, and antioxidant defense system, and it is closely related to the occurrence of ferroptosis. Heat stress downregulates GSH synthesis and inhibits antioxidant responses, further aggravating ROS damage [28]. Collectively, the tissue or cell damage caused by HS-induced oxidative stress is likely to be associated with ferroptosis. This study found a decrease in GPX4 expression and GSH production while an increase in iron levels, malondialdehyde (MDA), and ROS accumulation in HS-induced ALI, suggesting that ferroptosis is indeed involved in HS-induced ALI.

It is confirmed that ferroptosis is one of the most important mechanisms underlying rhabdomyolysis in cases of exertional HS [29]. Interestingly, this finding has also been observed in plants in which Arabidopsis root hair cells to heat shock develop cellular ferroptosis [30]. Ferroptosis, therefore, may play an important role in the pathophysiological processes of HS. The study of Li et al. reported that panaxydol attenuated ferroptosis in mice with lipopolysaccharide (LPS)-induced ALI via the Keap1-Nrf2/HO-1 pathway [31]. Guo et al. found that salidroside attenuated HALI via IL-17A-mediated ferroptosis in alveolar epithelial cells by regulating the Act1-TRAF6-p38 MAPK pathway [14]. Li et al. found that activation of the P62-Keap1-Nrf2 pathway protected against ferroptosis in radiation-induced lung injury [32]. The study of Li et al. demonstrated that apoptosis-stimulating protein p53 inhibitor inhibited ferroptosis and alleviated intestinal ischemia/reperfusion-induced ALI [33]. Taken together, ferroptosis plays a crucial role in the development of ALI of different etiologies. Thus, this study aimed to find a way to protect against HS-induced ALI from the aspect of ferroptosis. Our previous study reported that ALA protected against sepsis-induced ALI mainly by inhibiting the expression of inflammatory factors [16,22]. It was observed that ALA significantly reduced pulmonary vascular hypertension, attenuated pulmonary edema, decreased pulmonary vascular resistance and permeability, and reduced the infiltration of inflammatory cells in the lung tissue. This study revealed that ALA reduced inflammatory response in HS-induced ALI. As reported, ALA can also inhibit oxidative stress, reduce LPO, promote antioxidant synthesis, and reduce apoptosis after oxidative stress [34,35]. In this study, the ALA + HS group demonstrated lower levels of iron, MDA, and ROS accumulation and higher levels of GPX4 expression and GSH in vivo, as compared to the HS group. In vitro, analysis showed lower ROS and LPO production in BMDMs in the ALA + HS group. These results suggest that ALA has a protective effect against HS-induced ALI.

The specific molecular mechanism by which ALA protects against HS-induced ALI by reducing ferroptosis was investigated in this study. Ferroptosis is an iron-dependent form of cell death. Different from apoptosis and necrosis, ferroptosis occurs when ROS levels are lethal [36]. LPO occurs when cells are under stress. Exposure to excessive ROS leads to an oxidative stress response, indicating that the body itself can produce a series of protective proteins to mitigate ROS damage to cells [37]. GPX4 is crucial in scavenging lipid peroxides. SLC7A11 mediates the uptake of extracellular cystine, an important raw material for intracellular biosynthesis of GSH [38]. Nrf2 is a central regulator of redox, metabolism and protein homeostasis, and it is rapidly degraded in the cytoplasm in normal physiological conditions. Under ROS attacks, Nrf2 rapidly translocates to the nucleus, where it binds to corresponding antioxidant response elements (AREs) and then triggers the transcription of target genes SLC7A11 and GPX4, thus exerting antioxidant effects [39,40]. Research reported that Ozone pretreatment alleviated ischemia-reperfusion injury-induced myocardial ferroptosis by activating the Nrf2/Slc7a11/Gpx4 axis [41]. Additionally, Kaempferol ameliorated oxygen-glucose deprivation/reoxygenation-induced neuronal ferroptosis by activating the Nrf2/SLC7A11/GPX4 axis [42]. It was also found that astragaloside IV regulated the ferroptosis-related signaling pathway via the Nrf2/SLC7A11/GPX4 axis to inhibit PM2.5-mediated lung injury in mice [43]. Another study reported that ALA reduced ROS production and nitric oxide synthase 2 (NOS2) but increased Nrf2 expression, in turn inhibiting oxidative stress [44]. In this study, ALA activated Nrf2 and promoted its translocation to the nucleus, increasing the transcriptional levels of SLC7A11 and GPX4, reducing ROS production, and eventually inhibiting ferroptosis. Moreover, Nrf2 knockout exacerbated HS-induced ALI, which could not be rescued by ALA.

This study confirmed that ALA protected against HS-induced ALI through inhibiting ferroptosis and reported the underlying mechanism. Nevertheless, this study has some limitations. (1) Recent research reports that extreme hyperthermia caused by prolonged exposure to high environmental heat can lead to excessive activation of ZBP1-dependent cell death pathways, including pyroptosis, apoptosis, and necroptosis [15]. This demonstrates that pyroptosis, apoptosis, and necroptosis are involved in HS, while the effect of ALA on other cell death pathways was not explored. (2) This study confirmed that ALA inhibited ferroptosis in macrophages via activation of the Nrf2/SLC7A11/GPX4 pathway, but how ALA interacts with Nrf2 was not explained. Further research on how ALA modulates Nrf2 and upstream molecules will be carried out. (3) This study focused on the effect of ALA on ferroptosis in macrophages due to a significant increase in macrophage infiltration in HS. However, other cell types involved in the effect of ALA on ferroptosis were not considered, which will be a potential direction for our future research.

## 5. Conclusions

**Figure 7.**
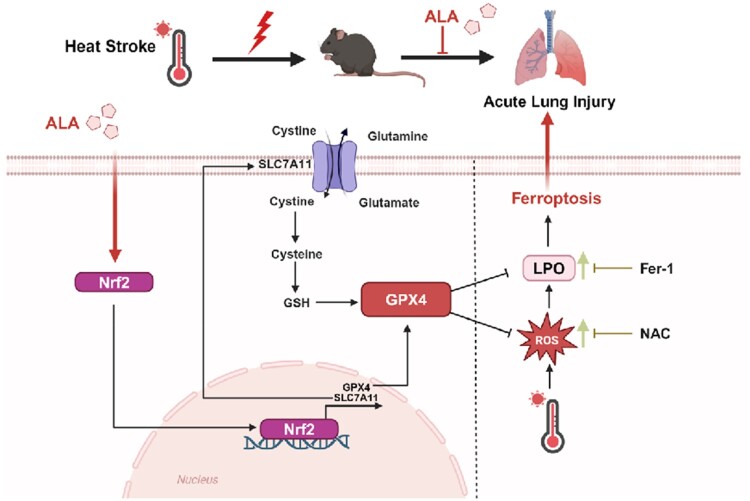
ALA protects against HS-induced ALI by reducing ferroptosis through the activation of the Nrf2/SLC7A11/GPX4 pathway. HS-induced ALI is characterized by increased ROS and LPO accumulation, decreased GSH production, and lower levels of GPX4. ALA attenuated ferroptosis in HS-induced ALI by promoting the translocation of Nrf2 into the nucleus, which enhances the expression of its downstream targets, GPX4 and SLC7A11 (created with BioRender.com).

Based on the findings of the study, a mechanistic model in which ALA attenuates HS-induced ALI is proposed (Figure 7). To conclude, ALA inhibited ferroptosis via activation of the Nrf2/SLC7A11/GPX4 signaling pathway, thereby attenuating HS-induced ALI. This study provides a new target for treating HS-induced ALI, and ALA may be a potential drug for treating HS-induced ALI.

## Author contributions

Conceptualization, L. W. and J. M.; methodology, J. M. and Z. L.; validation, X. Z., Y. L. and Y. C.; formal analysis, L. W., J. M. P. W and X. Y.; writing-original draft preparation, L. W., Z. L. and Y. C.; writing-review and editing, J. M., L. Y. and J. L.; funding acquisition, J. L. All authors have read and agreed to the published version of the manuscript.

## Supplementary Material

Supplementary_Materials_2.docx

Supplementary_Materials_1.docx

## Data Availability

The original contributions presented in the study are included in the article/supplementary material, and further inquiries can be directed to the corresponding authors.
