# Supplementary material for: Alpha-linolenic acid protects against heatstroke-induced acute lung injury by inhibiting ferroptosis through Nrf2 activation
Source: Redox Rep. 2025 Jul 27;30(1):2538294. doi: 10.1080/13510002.2025.2538294 (PMC12305878; doi:10.1080/13510002.2025.2538294)

Supplementary Materials

Figure S2. Genetest of *Nrf2^-/-^* mice. The homozygous *Nrf2^-/-^* mice presented a single band of 451 bp, the heterozygous *Nrf2^-/-^* mice presented two bands of 451 bp and 513 bp, and the wild-type allele presented a single band of 513 bp.


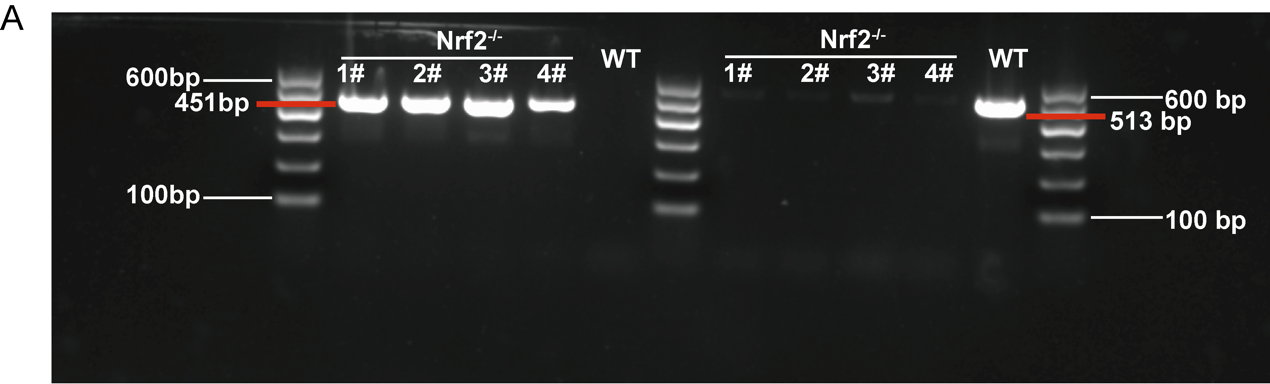

Supplement: Supplementary_Materials_2.docx [file YRER_A_2538294_SM2964.docx]
