# Supplementary material for: Alpha-linolenic acid protects against heatstroke-induced acute lung injury by inhibiting ferroptosis through Nrf2 activation
Source: Redox Rep. 2025 Jul 27;30(1):2538294. doi: 10.1080/13510002.2025.2538294 (PMC12305878; doi:10.1080/13510002.2025.2538294)

Supplementary Materials

Figure S1. Effects of a model of HS on survival and organs in mice. (A) Effects of different severities of HS on survival in mice. (B) Representative images showing H&E staining of multiple organs at different points after HS. Yellow arrowheads indicate edema, red indicates erythrocyte exudation, blue indicates inflammatory cell infiltrate, black indicates lumen expansion (scale bar = 200 μm), asterisk indicates loss of brush border, green indicates villous blunting and denudation (scale bar = 100 μm).


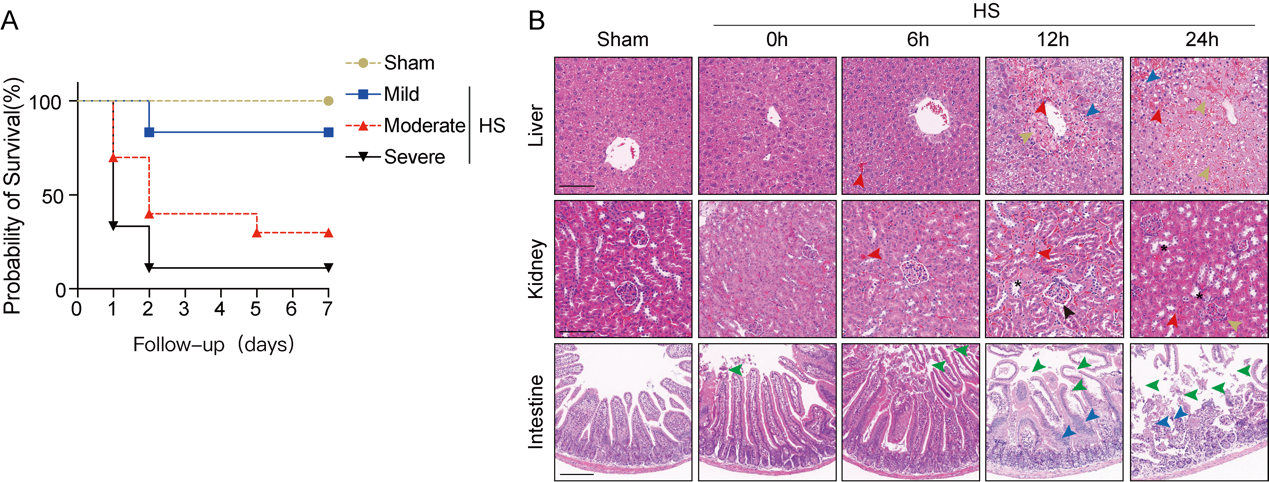

Supplement: Supplementary_Materials_1.docx [file YRER_A_2538294_SM2963.docx]
